# Economic evaluation of a single-pill triple antihypertensive therapy with valsartan, amlodipine, and hydrochlorothiazide against its dual components

**DOI:** 10.1186/s12962-015-0036-x

**Published:** 2015-06-09

**Authors:** Panagiotis Stafylas, Georgia Kourlaba, Magda Hatzikou, Dimitrios Georgiopoulos, Pantelis Sarafidis, Nikolaos Maniadakis

**Affiliations:** Medical Research & Innovation L.P., Adrianoupoleos 1, Kalamaria, Thessaloniki, GR-55133 Greece; Collaborative Center for Clinical Epidemiology and Outcomes Research, Athens, Greece; Novartis Hellas, Athens, Greece; Novartis UK, London, UK; Department of Nephrology, Aristotle University, Thessaloniki, Greece; National School of Public Health, Athens, Greece

**Keywords:** Hypertension, Blood pressure, Valsartan, Amlodipine, Cost-effectiveness, Cost-utility, QALY

## Abstract

The aim of this study was to compare the cost-utility of the first available single-pill triple combination antihypertensive therapy containing valsartan (V), amlodipine (A) and hydrochlorothiazide (H), with each of the same components dual combinations in patients with moderate to severe hypertension.

A Markov model with eight health states was constructed. The short-term effect of antihypertensive treatment on blood pressure was extrapolated through the Hellenic SCORE and Framingham risk equations, estimating the long-term survival and quality-adjusted life-years (QALYs) saved. Costs and outcomes were evaluated over lifetime, divided into annual cycles and discounted at 3.0 % with 2013 as reference year. The analysis was conducted by the Greek third-party-payer perspective.

The triple combination treatment cost was estimated at €16,525 compared to €15,480 for V/A, €14,125 for V/H and €11,690 for A/H. The QALYs saved with the triple combination were 12.76 vs. 12.64, 12.61 and 12.38 for double combinations respectively. The incremental cost-effectiveness ratio of the triple combination versus V/A and A/H was far lower than the Greek GDP per capita (€8,690/QALY and €12,695/QALY, respectively) and really close for V/H (€16,192/QALY), suggesting V/A/H combination to be cost-effective. Extensive sensitivity analyses confirmed the robustness of the results. The probability that the triple combination is cost effective was more than 90 % at a willingness-to-pay threshold of €18,000/QALY.

This is the first study to evaluate the cost-utility of a single-pill triple combination. The single-pill V/A/H therapy is a cost-effective antihypertensive choice for the treatment of moderate to severe hypertension, compared to its dual components.

**Electronic supplementary material:**

The online version of this article (doi:10.1186/s12962-015-0036-x) contains supplementary material, which is available to authorized users.

## Introduction

Hypertension is the most common chronic disease in the Western world; its prevalence in the adult population is higher than 25 %, and is estimated to increase by 30 % by 2025 [1, 2]. Furthermore, hypertension is a major risk factor for cardiovascular disease (CVD) (including coronary artery disease, heart failure and stroke), chronic kidney disease (CKD) and overall mortality [3–5]. Thus, elevated blood pressure (BP) is characterized as the primary attributable risk for death worldwide, accounting for approximately 7.5 million deaths (12.8 % of the total of all deaths) and 57 million disability-adjusted life years (DALYs) according to the World Health Organization (WHO) [6]. Extensive evidence suggests that antihypertensive treatment significantly reduces cardiovascular morbidity and mortality. At the same time, co-ordinated efforts in different countries have led to significant improvements in hypertension awareness and treatment rates; however, BP control rates remain low and unacceptable, particularly in Europe [3].

Several reasons for this poor control of hypertension have been proposed, among which inappropriate treatment regimens (i.e., absence of combination treatment, inadequate doses or inappropriate combinations) and poor adherence to treatment appear to be the most important [3, 7]. Therefore, current guidelines recommend immediate initiation of drug treatment in individuals with grade 3 hypertension alone or with grade 2 hypertension combined with 3 or more additional risk factors simultaneously with lifestyle changes [3]. Initiation of combined drug treatment and prompt up-titration is advisable for individuals with high CVD risk. When three drugs are required, the most rational combination appears to be a renin–angiotensin system blocker, a dihydropirydine calcium antagonist, and a diuretic at effective doses [8]. Furthermore, since regimen complexity is a major reason for poor compliance [7, 9], current recommendations advocate the use of fixed-dose single-pill combinations to improve BP control rates [3].

In November 2009, the European Medicines Agency (EMEA) approved the first single-pill, triple combination of amlodipine (A), valsartan (V) and hydrochlorothiazide (HCTZ, H), and several months later another triple combination with olmesartan, amlodipine and HCTZ was approved. The combination of V/A/H has shown [10] to be particularly potent and safe in moderate and severe hypertension. In Greece, where 25 %-30 % of the adult population are hypertensive, but less than one third of them are controlled [11], V/A/H constitutes, at the time this study was conducted, the first available single-pill triple antihypertensive combination but also the most expensive antihypertensive pill in the Greek pharmaceutical market.

Greece among other countries, especially in Southern Europe, is experiencing a very difficult economic situation and there are various austerity measures that will be implemented in the pharmaceutical sector. These measures aim to reduce the country’s annual pharmaceutical bill to less than 2 billion Euros. As the cost-effectiveness of this new V/A/H combination is totally unknown, there may be some arguments about whether it should be reimbursed by third-party-payers. Therefore, the aim of this study was to evaluate the cost-effectiveness of the single-pill combination V/A/H against each of the dual combinations deriving from the same components in patients with moderate to severe hypertension.

## Methods

A Markov model evaluating the cost-utility and the cost-effectiveness of the single-pill triple antihypertensive therapy V/A/H against each of the dual components was constructed. The short-term effect of antihypertensive treatment on BP was extrapolated through the use of predictive modelling in order to estimate the long-term survival [12]. The analysis was conducted by the Greek third-party-payer perspective, the recently founded National Organization for the Provision of Health Services (EOPYY), which covers the health expenditures of more than 90 % of Greek citizens. An annual discounting rate of 3 % was used for effectiveness and cost estimations [13].

### Overview of model structure

**Fig. 1:**
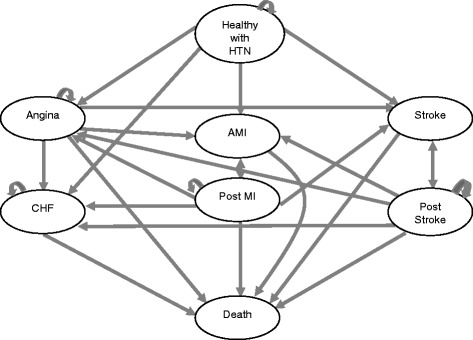
A graphical presentation of the Markov model

The structure of the model is illustrated in Fig. 1. In particular, this model consists of eight states of health status in respect of cardiovascular morbidity and mortality: “Healthy” with hypertension, angina, acute myocardial infarction (AMI), post AMI, stroke, post-stroke, congestive heart failure (CHF) and death from cardiovascular or other cause. A particular cost and quality of life (QoL) value is assigned to each health state. Each arrow represents a transition from one health state to another, which can occur with a certain probability at yearly intervals. In general, patients enter the model in the “healthy with hypertension” state and can experience one of the following events during each model cycle: death, angina, AMI, stroke or CHF. Patients experiencing angina can move into AMI, stroke, CHF or death. Patients experiencing a CHF event can only die during the following model cycles; otherwise they remain in the same state. Patients experiencing an AMI or stroke spend one year in an interim state, before transitioning to the post-AMI or post-stroke state. Only patients that do not die during this first year make a transition to the “Post MI” and “Post stroke” states. These states are very similar to the “AMI” and “stroke” states. However, the main idea behind this specific construct of the Markov model is to allow for a worse prognosis and higher cost in the first year after a non-fatal event compared with the second and subsequent years. Patients at the state of “post-MI” (or “post-stroke”) may experience a recurrent event and then transit to the AMI state (or “stroke”), or experience another non-fatal (i.e., angina, CHF, stroke) or fatal event. The model runs up to the time that it can be assumed that all patients are dead. The model was developed in Microsoft Excel 2003.

### Model inputs

#### Comparators, doses and treatment effect

In the present study the single-pill triple treatment of V/A/H 320/10/25 mg was compared with each of the dual components: V/H 320/25 mg, V/A 320/10 mg and A/H 10/25 mg. The treatment measure was the mean reduction in systolic blood pressure (SBP), which was extracted from the only head-to-head clinical trial published in 2009 demonstrating a 39.7, 32.0, 33.5 and 31.5 mmHg reduction for the V/A/H, V/H, V/A and A/H combinations respectively (Additional file 1: Figure S1) [14].

#### Transition probabilities

The probabilities for experiencing a fatal or non-fatal cardiovascular event (transition probabilities) were calculated based on the post-treatment blood pressure, which is incorporated in the risk equations of Hellenic SCORE and Framingham, along with other relevant parameters. The Hellenic SCORE risk equation was used to calculate the 10-year cardiovascular death risk, which was subsequently transformed into the corresponding annual probability [15]. The Framingham risk equation was used to calculate the annual probability of experiencing an AMI, angina, CHF, stroke as well as subsequent non-fatal AMI and angina [10, 16–19]. The annual probability for a subsequent stroke event was extracted from the most recent NICE guideline on hypertension [20] due to lack of relative predictive equations. Transition probabilities for non-cardiovascular death were estimated by using the Greek mortality rates based on the latest data of the Hellenic Statistical Authority [21].

#### Baseline population

The demographic and baseline characteristics of the target population are described in Table 1. It was assumed that the Greeks had a low cardiovascular risk profile based on epidemiological data [22–24]. The “High” and “Low” risk refer to the two risk regions of the European population, which exhibit the highest and lowest risk for developing cardiovascular diseases. The former includes countries such as Russia, Bulgaria and Latvia, while the latter includes countries such as Greece, Sweden, France and Germany [22].

**Table 1:** Summary of demographic and clinical input parameters applied in the baseline model

Characteristic	Value	Reference
Mean age (SD), years	53.2 (10.3)	Calhoun et al. (2009) [10]
Male (%)	55.3	Calhoun et al. (2009) [10]
Mean SBP (SD), mmHg	169.9 (14.1)	Calhoun et al. (2009) [10]
Mean DBP (SD), mmHg	106.5 (5.1)	Calhoun et al. (2009) [10]
Male smokers (% of males)	51 %	Pitsavos et al. (2003) [23]
Female smokers (% of females)	39 %	Pitsavos et al. (2003) [23]
Male diabetics (% of males)	11.2 %	WHO (accessed 11/2013) [24]
Female diabetics (% of females)	10.5 %	WHO (accessed 11/2013) [24]
Total cholesterol (mmol/L)	5	Pitsavos et al. (2003) [23]
HDL cholesterol (mmol/L)	1.25	Pitsavos et al. (2003) [23]
Body mass index (kg/m^2^)	27	Pitsavos et al. (2003) [23]

*Abbreviations*: *SBP* systolic blood pressure, *DBP* diastolic blood pressure

#### Utility values

Due to lack of utility values for the Greek population under evaluation, health utilities were extracted from recent published studies and reviews that have already been applied to similar Greek populations [25]. QALYs were calculated based on the above utility weights, whereby the assumption applied was that patients experiencing an event have constant utility for the whole duration of one year (Table 2).

**Table 2:** Utilities used in the model (Maniadakis et al. 2011 [25])

Age	Healthy/HTN		Angina/AMI/Post AMI		Stroke/Post-stroke		Heart Failure	
	Men	Women	Men	Women	Men	Women	Men	Women
20	0.91	0.88	0.84	0.81	0.82	0.79	n.a.	n.a.
25	n.a.	n.a.	n.a.	n.a.	n.a.	n.a.	0.69	0.63
30	0.90	0.86	0.83	0.79	0.81	0.77	n.a.	n.a.
40	0.86	0.85	0.79	0.78	0.77	0.76	n.a.	n.a.
46	n.a.	n.a.	n.a.	n.a.	n.a.	n.a.	0.61	0.56
50	0.84	0.82	0.77	0.75	0.75	0.73	n.a.	n.a.
60	0.83	0.78	0.76	0.71	0.74	0.69	n.a.	n.a.
65	n.a.	n.a.	n.a.	n.a.	n.a.	n.a.	0.57	0.52
70	0.81	0.78	0.74	0.71	0.72	0.69	n.a.	n.a.
80	0.74	0.74	0.67	0.67	0.65	0.65	n.a.	n.a.

*Abbreviations*: *AMI* acute myocardial infarction, *HTN* hypertension, *N/A* non-applicable

#### Costs calculation

In the present model, only direct health care costs were taken into consideration. The total state-specific cost was calculated based on the costs of hospitalisation, outpatient visits, concomitant medications, laboratory and imaging diagnostic examinations (Table 3) as well as the cost of antihypertensive treatment (Table 4). The cost derived from the treatment of adverse events was not considered in this analysis because there were no statistical differences among the different treatment choices [10]. The hospitalisation cost was estimated based on the Diagnostic Related Groups (DRGs). This cost was obtained from the Government Gazette issued in March 2012 by the Ministry of Health. The rest of the cost components were calculated by multiplying the number of units for each resource (i.e., number of outpatient visits) by the corresponding unit cost available from the Government Gazette. The unit costs data correspond to the year 2013. For the estimation of patient-level antihypertensive medication costs, the daily dose of each drug was combined with the relevant price obtained from the bulletin issued by the Greek Ministry of Health. The annual cost of each comparator medication was calculated based on the assumption that patients would take the same daily dose of each drug, every day throughout the year, until they experience a fatal event.

**Table 3:** Cost of health resources used in the baseline model (in Euros, €)

Hospitalisation costs^a^	DRG code	Cost
Condition		
Hypertension	K37X	355
Angina	K47M	940
Post-Angina	K47X	424
Myocardial Infarction (MI)	K40M	1818
Post-MI	K32X	968
Stroke	N30Mβ	1625
Post-Stroke	N30Mβ	1625
CHF	Κ42Χ	849
Rehabilitation (Stroke)	S20Χ	2716
Death	-	1200
Intervention		
PCI	Κ15Χ	1761
CABG	Κ05Χ	6495
Angiography	Κ32Α	498
Pacemaker	Κ12Χ	2831
ICD	Κ01Χ	11,291
CRT	-	7270
Heart Transplantation	Ε05Α	34,000
Clinical, Laboratory and Imaging Exam Costs^b^		Cost
Outpatients Visits		10
Physiotherapists		20
CT Scans		71.11
MRI		236.95
MRA		235.00
Thallium Scintigraphy		260.00
Carotid Triplex		73.37
Echocardiography		70.00
Blood and Biochemistry Tests^c^		57.40
Treadmill Stress Test		28.11

*Abbreviations*: *CHF* congestive heart failure, *PCI* percutaneous coronary intervention, *CABG* coronary artery bypass graft, *ICD* implantable cardioverter-defibrillator, *CRT* cardiac resynchronisation therapy, *CT* computed tomography, *MRI* magnetic resonance imaging, *MRA* Magnetic Resonance Angiography

^a^Hospitalisation costs derive from the Government Gazette issued in March 2012 by the Ministry of Health; cost of death is extracted from Maniadakis et al. 2005 [37] and CRT cost is obtained from a Government Gazette published in 2007

^b^Clinical, laboratory and imagining examination costs are obtained from a Government Gazette that is valid in November, 2013

^c^Routine laboratory tests include blood count, urine test, test for levels of glucose, urea, creatinine, sodium, potassium, SGOT (serum glutamic-oxaloacetic transaminase), SGPT (serum glutamic-pyruvic transaminase), CPK (creatine phosphokinase), total cholesterol, HDL-C (high-density lipoprotein cholesterol), LDL-C (low-density lipoprotein cholesterol), triglycerides, uric acid

**Table 4:** Drug prices^a^ used in the baseline model (in Euros, €)

Drug name	Package (tablets × dose in mg)	Retail price per package	Daily cost for EOPPY
V/A/H branded^b^	28 × 320/10/25	43.84	1.17
V/A branded^b, c^	28 × 160/5	26.50	1.42
V/H branded	14 × 320/25	14.42	0.77
V/H generic	14 × 320/25	10.96	0.59
Valsartan branded	14 × 320	11.77	0.63
Valsartan generic	28 × 320	13.86	0.37
Amlodipine branded	14 × 10	6.78	0.36
Amlodipine generic	30 × 10	9.69	0.24
Hydrochlorothiazide	20 × 25	0.69	0.03

^a^Drug prices were obtained through the Government Gazette published by the Greek Ministry of Health and are expressed in 2013 values (€)

^b^No available generic for V/A/H and V/A combinations; not available A/H combination neither in branded nor generic form

^c^No available package at 320/10 mg; therefore two tablets per day were necessary to reach the same strength

### Economic analysis

The primary outcome was the cost-utility of the triple antihypertensive treatment over comparators, calculating the incremental cost effectiveness ratio (ICER) of Euros per quality-adjusted life years (QALY) saved. Secondary outcomes included QALYs saved, life-years gained (LYG), total cost for each comparator and the ICER per LYG. In general, when V/A/H is associated with higher effectiveness and higher cost, it is considered highly cost-effective when the ICER is lower than the Gross Domestic Product (GDP) per capita of the country, cost-effective when the ICER is one to three times higher than the GDP per capita and non cost-effective when the ICER is higher than three times the GDP per capita – a specific predetermined threshold recommended by the World Health Organization [26, 27]. According to the International Monetary Fund (IMF), the GDP per capita in Greece has been estimated at €16,303 for 2013 [28].

The majority of input data used in the current model are subject to variation. Therefore, in order to overcome the issue of uncertainty, a probabilistic sensitivity analysis was conducted. Probability distributions were selected based on the nature of variables [29]. For costs, the logarithms were assumed to be normally distributed. In general, the distribution parameters were estimated based on mean and standard deviations (SD) of published data (if available), whereas in case information on parameters variability was unavailable, their standard deviation was assumed to be equal to 10 % of the mean.

Following this, 5000 estimates of costs, LYs, QALYs, and incremental cost per QALY saved and per LYG (life year gained) were obtained by applying the bootstrapping technique. The bootstrap percentile method was used to obtain the uncertainty appropriate intervals for each parameter, as described elsewhere [30]. The results are presented as mean (95 % uncertainty interval). A cost-effectiveness acceptability curve was plotted, indicating the proportion of simulations according to which V/A/H is considered cost-effective over its comparators at different levels of willingness to pay per effectiveness unit gained [31].

#### Sensitivity analyses

In our study, the impact of six assumptions used in the Markov model was tested in one-way sensitivity analyses by varying the following data: (1) costs and outcomes were discounted at 0 % and at 6 %, (2) drugs were substituted by the cheapest generics, (3) the costs and the use of other health resources were modified (±50 %), (4) drug prices were estimated based on alternative sickness fund coverage, and the synthesis of the population was changed to include (4) patients with different age groups (40, 60 and 70 years), (5) 100 % patients with diabetes, and (6) 100 % patients with prior CVD.

## Results

### Costs

The total discounted cost for participants receiving the triple combination (V/A/H) was €16,525.25, whereas for the dual combinations of V/H, V/A and A/H the total discounted costs were €14,124.74, €15,480.46, €11,690.08 respectively (Table 5). In all combinations, except for A/H, total costs were made up mainly of drug costs, which accounted for 41.54 %, 31.60 % and 37.21 % of the overall costs of V/A/H, V/H, V/A respectively.

**Table 5:** Total Costs per Category (values in Euros (€)

Cost Category	V/A/H	V/H	A/V	A/H
Drugs	6863.94	4464.02	5760.68	2206.07
Other Medication	1732.86	1733.97	1773.32	1690.92
Hospitalisation	2567.05	2613.56	2621.59	2560.13
Outpatient Visits	400.32	402.42	403.43	394.44
Vascular Interventions	1818.07	1779.99	1785.88	1743.21
Laboratory Exams	2572.37	2552.66	2558.97	2501.37
Death	570.62	578.11	576.59	593.94
Total Cost^a^	16,525.25	14,124.74	15,480.46	11,690.08

^a^Adding up the values for each combination might differ from the total cost value due to rounding

### Clinical outcomes

Table 6 presents the effectiveness results comparing the triple single-pill combination (V/A/H) with each possible dual combination. The analysis showed that the triple combination was more effective compared to its dual components for both estimates of QALYs and LYGs. Patients who received V/A/H gained 12.76 QALYs and 15.99 LYs. Therefore, V/A/H was associated with a gain of 0.12 to 0.38 QALYs and 0.14 to 0.49 LYs when compared with its dual components.

**Table 6:** Baseline deterministic cost, effectiveness and cost-effectiveness results comparing the triple single-pill combination (V/A/H) with each possible dual combination

Treatment alternative	Cost (€)	Incremental Cost (€)	Effectiveness (QALYs/LYs)	Incremental effectiveness (QALYs/LYs)	ICER (€/QALY or €/LY)
*In QALYs*					
V/A/H	16,525.25		12.76		
V/H	14,124.74	2400.51	12.61	0.15	16,192.40
V/A	15,480.46	1044.79	12.64	0.12	8690.13
A/H	11,690.08	4835.17	12.38	0.38	12,694.89
*In LYs*					
V/A/H	16,525.25		15.99		
V/H	14,124.74	2400.51	15.81	0.18	7456.95
V/A	15,480.46	1044.79	15.85	0.14	4073.82
A/H	11,690.08	4835.17	15.50	0.49	5002.20

*Abbreviations*: *V* valsartan, *H* hydrochlorothiazide, *A* amlodipine, *QALY* quality-adjusted life year, *LY* life year, *ICER* incremental cost-effectiveness ratio

### Cost-effectiveness

The ICER was calculated with respect to the triple single-pill combination of V/A/H (Table 6). Compared to the examined dual combinations, V/A/H presented a cost per QALYs ratio of €16,192.40 when tested against V/H combination, of 8690.13 against V/A combination and of €12,694.89 against the A/H combination.

**Fig. 2:**
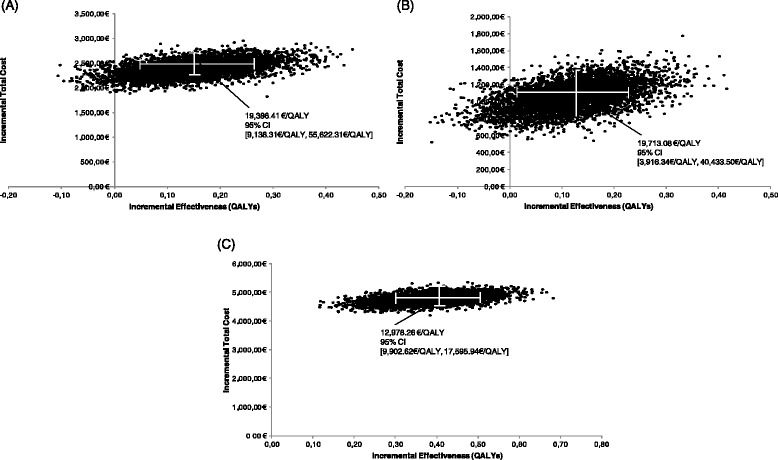
Cost-effectiveness plane for V/A/H versus V/H (**a**), V/A (**b**) and A/H (**c**). Εach point represents an estimate of the ICER in €/QALY

**Fig. 3:**
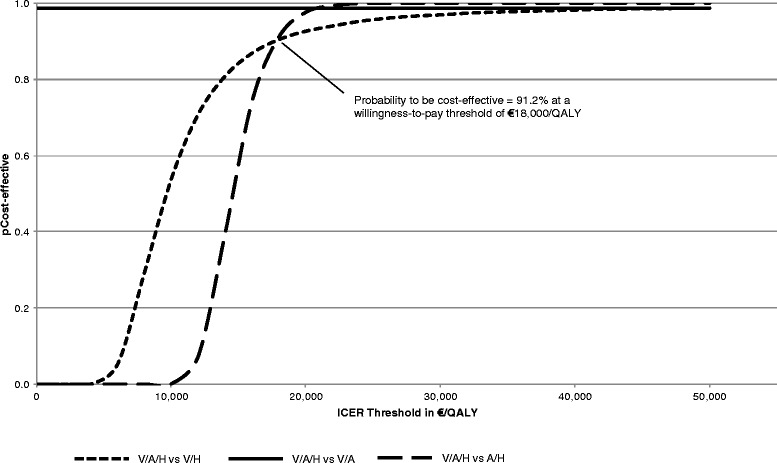
Cost-effectiveness acceptability curve

The probabilistic analysis also suggested that V/A/H is cost-effective compared to V/H, V/A and A/H dual combinations, since the mean ICER is either slightly higher or lower than the GDP per capita. For patients receiving the triple combination, there was an incremental survival benefit (in QALYs) of 0.15 (95 % CI, 0.04-0.26), 0.12 (95 % CI, 0.01-0.23) and 0.38 (95 % CI, 0.27-0.49) compared to V/H, V/A and A/H combinations respectively (Figs. 2a, b, c). Based on the above data, there is more than 90 % probability for the triple combination to be cost-effective at a willingness-to-pay threshold of €18,000/QALY (Fig. 3).

### Sensitivity analyses

Table 7 depicts our sensitivity analyses. Our results were shown to be robust for the six variables we evaluated in one-way sensitivity analyses. In most cases, the single-pill combination was proven to be a cost-effective choice, since the ICER value was lower than €20,000/QALY and usually lower than the GDP per capita.

**Table 7:** One-way sensitivity analyses (Incremental Cost-Effectiveness Ratio expressed in €/QALY; discounted at 3 %)

Value	V/H	V/A	A/H
Baseline	16,192.40	8690.13	12,694.89
Discount rate			
Low: 0 %	9136.23	4899.12	6528.41
High: 6 %	18,887.19	10,127.14	15,888.91
Generic Substitution			
Cheapest generics	30,021.13	21,881.33	12,870.17
Marginal price (further price decrease −75 %)	41,707.74	51,015.44	17,685.29
Health resources cost			
50 % decrease	16,188.30	8766.81	12,518.28
50 % increase	16,202.98	8620.55	12,875.81
Price estimation based on alternative sickness fund coverage			
Deterministic	30,021.13	15,609.78	11,069.45
Probabilistic (mean)	26,105.42	18,295.66	11,298.31
Age group			
40 years	12,976.54	7133.45	16,440.77
60 years	19,162.85	10,037.84	11,961.30
70 years	23,846.40	11,883.61	9960.84
Clinical characteristics			
All patients diabetics	16,477.38	8711.76	13,137.42
Prior CVD	15,342.43	7966.95	11,985.83

*Abbreviations*: *V* valsartan, *H* hydrochlorothiazide, *A* amlodipine, *CVD* cardiovascular disease

In the case of generic substitution, although the single-pill triple combination remained more costly (due to drug costs) compared to the other dual combinations, it was still cost-effective in comparison with the cheapest generic combinations. By examining fluctuations in the prices of generics, we determined that V/A/H ceases to be cost-effective only in the case of a generic drug price reduction of 75 % and above.

## Discussion

The present study is the first cost-effectiveness analysis of a single-pill triple antihypertensive combination versus its dual components. This analysis was performed from the perspective of the Greek third-party payer, which is currently covering the healthcare expenditures of more than 90 % of the Greek population. The clinical effectiveness of all comparators was based on the results of the only head-to- head clinical trial available to date [14]. Using a Markov model and assuming that the beneficial effects of the triple combination and the respective comparators would be maintained while patients remain on these drugs, we estimated that the triple single-pill combination would be expected to increase life expectancy by 0.14 to 0.49 years and QALYs by 0.12 to 0.38 in comparison with its dual components. We also observed that the ICER for V/A/H in the main analysis in comparison with the three dual combinations under examination (A/H, V/H and V/A) was lower than the Greek GDP per capita. These results suggest that although the triple combination is the most expensive antihypertensive pill in Greece, it is also a cost-effective choice among patients with grade 2 and 3 hypertension.

In the present study, we also conducted a probabilistic analysis and extensive sensitivity analyses modifying the most important input parameters in order to test the robustness of our results. There was a probability of more than 90 % for the triple combination to be cost-effective at a willingness-to-pay threshold of €18,000/QALY. The results of these analyses were particularly stable in modifying the input parameters regarding the prevalence of diabetes and the incidence of prior CVD. The results remained constant in different age groups; however the ICER was lower in younger hypertensive patients reflecting the higher lifetime risk of cardiovascular adverse events and the importance of hypertension control in younger ages. The modification of the health resources cost (±50 %) did not change the results significantly, nor did the estimation of the daily drug cost for the third-party payer by using an alternative sickness fund coverage method that has recently been introduced and is based on the following formula: Daily Drug Cost = [75 % (Sickness Fund Price)] – [50 % (Retail Price – Sickness Fund Price)]. Finally, the substitution of the branded drugs by the cheapest available generics maintained the cost-effectiveness of the triple combination, despite the increase in the ICER values. The analysis showed that for V/A/H not to be cost-effective, generic prices should fall further by at least 75 %.

Suboptimal antihypertensive regimens, including absence of treatment intensification or inappropriate combinations have long been considered a main reason for the poor control rates of hypertension in many countries [3, 7]. It has also been documented for a long time that a large proportion of patients presenting treatment-resistant hypertension, in reality, either receives improper combinations or is poorly compliant with the prescribed treatment [32]. For patients requiring more than two drugs to be controlled, a combination of a renin–angiotensin system blocker, a dihydropyridine calcium antagonist, and a diuretic at effective doses is widely proposed as the first choice due to proven efficacy and favorable adverse-effect profile [7, 8].

In addition to the above, it is well-known that adherence to antihypertensive treatment may fall by as much as 50 % within the first year [33], and regimen complexity is a major reason for this [7, 9]. Increased adherence to antihypertensive treatment is associated with better BP control [34] and reduced cardiovascular events [35]. Use of fixed-dose, single-pill combination treatment has recently been shown to markedly improve patient compliance [36], and thus, current recommendations advocate the use of fixed-dose combinations (including triple combinations, when necessary) to improve BP control and reduce associated morbidity and mortality [3]. Hence, it is of major importance to stress that this study probably underestimates the cost-effectiveness of the triple single-pill combination, as it is expected that a single-pill formulation would further improve adherence and persistence to treatment, thus leading to better BP control and reduced cardiovascular complications, versus comparator treatments including 2 to 3 different pills.

There are some potential limitations to our study. Since transition probabilities specific to the Greek population were not available, probabilities for AMI, angina, CHF, stroke, subsequent non-fatal AMI and angina were extracted from the Framingham study and probabilities for subsequent stroke and hypertension from the relevant NICE guidelines. The utility values used are also not specific to the Greek hypertensive population and a divergence affecting the outcomes of the model could be possible. We strongly believe, however, that no apparent epidemiological reasons exist for the Greek hypertensive population to differ from the relevant population of other Western Societies in the above-mentioned parameters.

To estimate the medication cost we assumed that patients remain on the same daily dose throughout the duration of the model; this might not always be the case, affecting the cost-effectiveness results. Finally, a limitation rather common to modelling analyses is the fact that QALY results are driven primarily by the cost of the drugs. We used a sensitivity analysis with generic substitution to address this issue and we found that drug cost has an impact on our results but did not affect the final cost-effectiveness profile of the triple single-pill combination over the dual combinations.

In conclusion, this first cost-effectiveness analysis of a triple single-pill antihypertensive combination suggests that a triple single-pill combination of valsartan, amlodipine and hydrochlorothiazide is expected to increase life expectancy and QALYs in comparison with each of the dual components, and it is also a cost-effective choice, as its ICER versus the dual combinations was lower than the Greek GDP per capita. These results were proven robust after an extensive set of sensitivity analyses. Additionally, the beneficial effects of increased compliance following a single-pill combination may in reality increase the cost-effectiveness of the triple combination. Further studies in different settings or with different triple single-pill combinations are awaited to shed more light on this field.

### Perspectives

Overall, the single-pill triple combination fulfills all the current guideline recommendations for patients with grade 2 and 3 hypertension or high cardiovascular risk as it is one of the most effective antihypertensive pills in reducing SBP and DBP, it allows early initiation of combination therapy and is the most rational combination, containing a blocker of RAAS, a calcium antagonist and a diuretic at effective doses. Moreover, it is the first available single-pill triple combination antihypertensive therapy and one of the three approved by the EMEA. The above in addition to the cost-effectiveness nature of the drug indicated by the results of our study, render the triple combination a preferable choice for the treatment of grade 2 and 3 hypertension.

### Novelty and Significance: 1) What Is New, 2) What Is Relevant?

1. What Is New?
  The present study constitutes the first cost-effectiveness analysis of a triple single-pill combination for the management of high blood pressure versus its dual components.
2. What Is Relevant?
  Recent treatment guidelines clarify the preference for single-pill combinations since they increase treatment adherence, improve patient compliance and therefore, reduce morbidity and mortality associated with inadequate blood pressure control. Therefore, it is of great importance to perform an economic evaluation for such a triple combination in order to examine its cost-effectiveness and determine whether it should replace current treatments or not. Due to the present economic situation, extra light should be shed on such a cost-effectiveness analysis as its results might lead to long-term cost-savings within healthcare budgets.
3. Summary
  The results of our study showed that the single-pill triple combination is cost-effective compared to its dual components, rendering it a preferable choice for the treatment of grade 2 and 3 hypertension.

## Additional file

Additional file 1: Figure S1. Changes from baseline in mean sitting systolic and diastolic blood pressure at the end of the 8-weeks follow-up (Calhoun et al. Hypertension 2009; 54: 32-39 [14]).
