# Supplementary material for: Economic evaluation of a single-pill triple antihypertensive therapy with valsartan, amlodipine, and hydrochlorothiazide against its dual components
Source: Cost Eff Resour Alloc. 2015 Jun 9;13:10. doi: 10.1186/s12962-015-0036-x (PMC4474457; doi:10.1186/s12962-015-0036-x)
Supplement: Additional file 1: Figure S1. — Changes from baseline in mean sitting systolic and diastolic blood pressure at the end of the 8-weeks follow-up (Calhoun et al. Hypertension 2009; 54: 32-39 [14]). [file 12962_2015_36_MOESM1_ESM.docx]

**Appendix**

**Figure. Changes from baseline in mean sitting systolic and diastolic blood pressure at the end of the 8-weeks follow-up (Calhoun et al. Hypertension 2009; 54: 32-39)**
